# ﻿A taxonomic review of the genus *Eversmannia* Staudinger, 1871 (Lepidoptera, Uraniidae) from China, with descriptions of three new species and three new combinations

**DOI:** 10.3897/zookeys.1251.145353

**Published:** 2025-09-10

**Authors:** Ming-Xu Han, Hui-Lin Han

**Affiliations:** 1 Northeast Forestry University, School of Forestry, Harbin, 150040, China Northeast Forestry University Harbin China; 2 Northeast Forestry University, Key Laboratory of Sustainable Forest Ecosystem Management-Ministry of Education, Harbin, 150040, China Northeast Forestry University Harbin China; 3 Northeast Forestry University, Northeast Asia Biodiversity Research Center, Harbin, 150040, China Northeast Forestry University Harbin China

**Keywords:** Epipleminae, key, swallowtail moth, taxonomy

## Abstract

Seven species of the genus *Eversmannia* Staudinger, 1871 are recognized from China. Among them, three species are described as new species: *E. atromarginata***sp. nov.**, *E. spiralis***sp. nov.**, and *E. zhangorum***sp. nov.** New combinations are proposed for three species: *E. bicaudata* (Moore, [1868]) **comb. nov.**, *E. himala* (Butler, 1880), **comb. nov.**, and *E. fuscifrons* (Warren, 1896), **comb. nov.***Eversmannia bicaudata* and *E. fuscifrons* are reported for the first time from China. Adults and genitalia are illustrated, and a key for identifying Chinese *Eversmannia* species is provided.

## ﻿Introduction

The genus *Eversmannia* was established as a monotypic genus from Kazan, Russia by Staudinger (1871). The type species *Idaea exornata* Eversmann, 1837 was originally mistakenly classified as belonging to Geometridae and placed in the genus *Idaea* Treitschke, 1825. Staudinger (1871) corrected this mistake and transferred it to the new genus *Eversmannia.* The distribution of *Eversmannia* is mainly in the Palearctic Region, with *E. exornata* recorded from Russia, Japan, and North Korea (Sohn and Yen 2005; Kishida 2011). In China, *E. exornata* was previously recorded in Heilongjiang Province (Chu et al. 2004).

In this study, we describe three new species, redescribe two newly recorded species from China, and transfer three species from *Epiplema* Herrich-Schäffer, 1855 to *Eversmannia* based on external and genital characters. This increases the number of species within *Eversmannia* to seven. Updated generic and species diagnoses, illustrations, a key to Chinese *Eversmannia* species, and distributional data are also provided.

## ﻿Materials and methods

Study specimens were collected in China, using 220V/450W mercury lamp and DC black (UV) light. Standard methods for dissection and preparation of the genitalia slides were used (Kononenko and Han 2007). The specimens were photographed using a Nikon Z6II camera; the genitalia slides were photographed using an Olympus photo-microscope and were stacked using Helicon Focus v. 7.6 and further processed in Adobe Photoshop CC2019. The holotype of *E. atromarginata* sp. nov. is deposited in the collection of Sun Yat-sen University, Guangzhou, China, and the paratypes are deposited in the collection of Northeast Forestry University, Harbin, China. Other specimens examined, including type specimens of *E. spiralis* sp. nov. and *E. zhangorum* sp. nov. are deposited in the collection of Northeast Forestry University.

### ﻿Abbreviations for institutional collections

**HGNU** Huanggang Normal University, Huanggang, China

**IZCAS** Institute of Zoology, Chinese Academy of Sciences, Beijing, China

**NEFU** Northeast Forestry University, Harbin, China

**NKU** Nankai University, Tianjin, China

**NHMUK** The Natural History Museum, London, United Kingdom

**SHNU** Shanghai Normal University, Shanghai, China

**SYSU** Sun Yat-sen University, Guangzhou, China

## ﻿Taxonomic account

### ﻿ Eversmannia

Taxon classificationAnimaliaLepidopteraUraniidae

Staudinger, 1871

D341DB37-21D4-556B-B6FD-F0E719D0BF39

Eversmannia Staudinger, 1871, in Staudinger & Wocke, Catalog der Lepidopteren des Europaeischen Faunengebiets: 159. Type species: Idaea exornata Eversmann, 1837. Type locality: Kazan, Russia.

#### Diagnosis.

In external appearance, this genus is similar to *Europlema* Holloway, 1998 but can be distinguished by the following characters: (1) male genitalia: uncus hook- or spoon-shaped; valva bar-shaped, editum-costa complex bifurcate; in *Europlema* uncus triangular or lanceolate and covered with hair, valva triangular, transtilla long and curved; (2) female genitalia: papillae anales conical or hoof-shaped; ductus bursae long and tubular, membranous; in *Europlema*, papillae anales flat and broad; ductus bursae very short, sclerotized.

#### Description.

***Adult*.** Head white; labial palpus up-curved; in some species male with plumose antenna, female antenna filiform. Thorax and tegula white. Forewing ground color white, with brown or black patches; subbasal line and postmedial line brown or black. Hindwing white, with brown or yellow patches; outer margin with short extension at Rs and M_3_ veins. Abdomen covered with white scales.

***Male genitalia*.** Uncus hook- or spoon-shaped, sclerotized. Gnathos trident- or spike-shaped. Tegumen triangular. Valva bar-shaped, covered with dense hair; costa long and strap-like, extending to cucullus. Editum-costa complex bifurcate, one branch short, finger-shaped, another long and bar-shaped. Juxta membranous, horseshoe-shaped. Vinculum V- or U-shaped. Saccus broad and V or U-shaped. Aedeagus cylindrical shaped, slightly curved or strongly curved; vesica with several spine-like cornuti.

***Female genitalia*.** Papillae anales conical or hoof-shaped, covered with short setae. Apophysis posterioris longer than apophysis anterioris. Ostium bursae flat, sclerotized. Ductus bursae membranous, slightly curved or spiral. Corpus bursae oval or elongate and drop-shaped, in some species with separate tubular and globular parts; usually with variously shaped signum, sometimes absent.

#### Distribution.

China, Russia, Japan, North Korea, Bhutan, Nepal, India, Bangladesh, Finland, Estonia, Latvia, Lithuania, Belarus.

### ﻿Key to the species of *Eversmannia* in China based on genitalia

**Table:** 

1	Ductus bursae spiral shaped	***E. spiralis* sp. nov. (Fig. 27)**
–	Ductus bursae straight	**2**
2	Uncus spoon-shaped	**3**
-	Uncus hook-shaped	**4**
3	Ductus bursae long and slender	***E. bicaudata* comb. nov. (Fig. 23)**
–	Ductus bursae short and wide	***E. himala* comb. nov. (Fig. 26)**
4	Aedeagus bow-shaped, strongly curved	**5**
–	Aedeagus cylindrical, slightly curved	**6**
5	Gnathos spine-shaped	***E. fuscifrons* comb. nov. (Fig. 18)**
–	Gnathos spear-shaped	***E. zhangorum* sp. nov. (Fig. 21)**
6	Corpus bursae with three signa	***E. atromarginata* sp. nov. (Fig. 22)**
–	Corpus bursae with one signum	***E. exornata* (Fig. 24)**

### ﻿ Eversmannia atromarginata sp. nov.

Taxon classificationAnimaliaLepidopteraUraniidae

F445463D-4DF7-591C-BE0D-1D81EF203CDB

https://zoobank.org/5BA27761-3AC8-48FB-A14A-9EEA39023175

**Figures 1–14.**
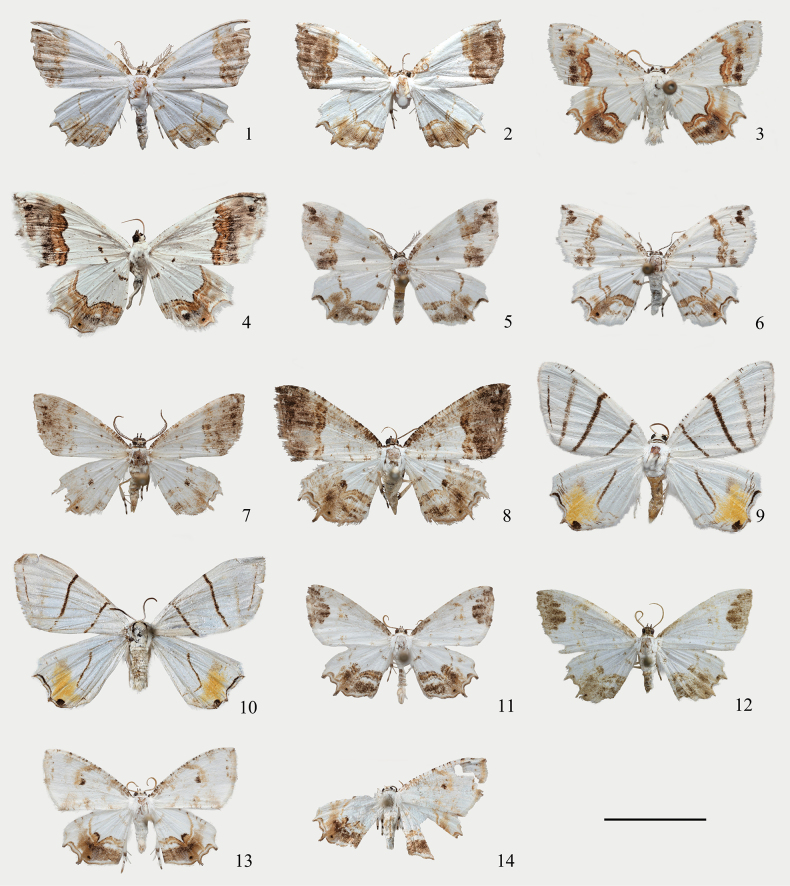
Adults of *Eversmannia* spp. (depositories of 1 in SYSU, 2–14 in NEFU). 1 *E. atromarginata* sp. nov., male, holotype (Jiangxi); 2 ditto, female, paratype (Sichuan); 3 *E. bicaudata* comb. nov., male (Xizang); 4 ditto, female (Xizang); 5 *E. exornata*, male (Heilongjiang); 6 ditto, female (Heilongjiang); 7 *E. fuscifrons* comb. nov., male (Xizang); 8 ditto, female (Xizang); 9 *E. himala* comb. nov., male (Sichuan); 10 ditto, female (Chongqing); 11 *E. spiralis* sp. nov., male, holotype (Chongqing); 12 ditto, female, paratype (Chongqing); 13 *E. zhangorum* sp. nov., male, holotype (Chongqing); 14 ditto, female, paratype (Guizhou). Scale bar: 1 cm.

**Figures 15–21.**
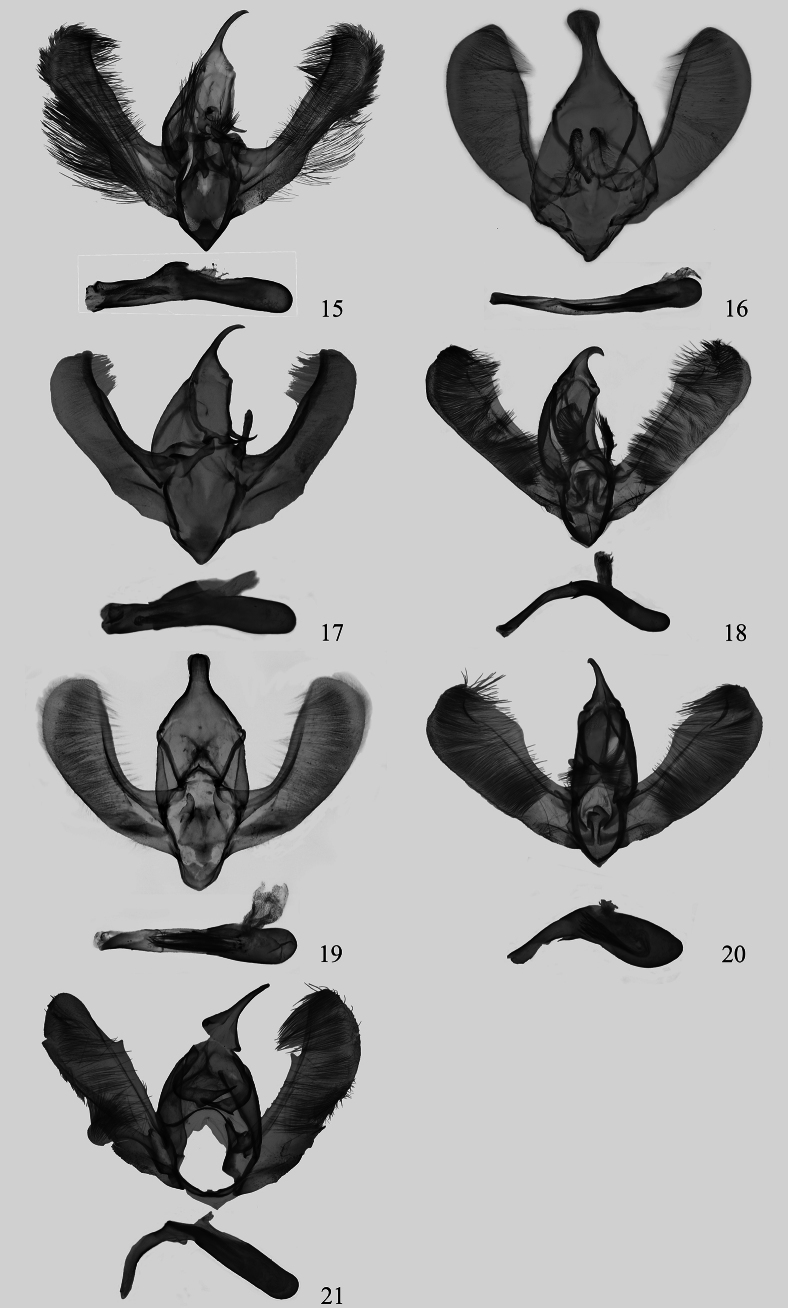
Male genitalia of *Eversmannia* spp. (depositories of all slides NEFU). 15 *E. atromarginata* sp. nov., paratype, slide hmx-232-1; 16 *E. bicaudata* comb. nov., slide hmx-141-1; 17 *E. exornata*, slide hmx-195-1; 18 *E. fuscifrons* comb. nov., slide hmx-216-1; 19 *E. himala* comb. nov., slide hmx-50-1; 20 *E. spiralis* sp. nov., paratype, slide hmx-250-1; 21 *E. zhangorum* sp. nov., holotype, slide hmx-249-1.

**Figures 22–28.**
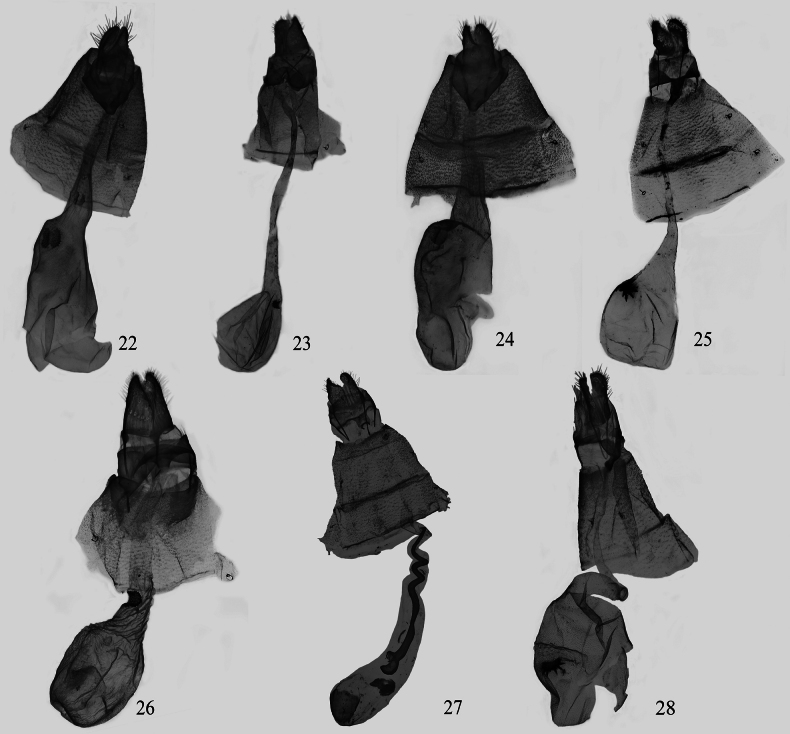
Female genitalia of *Eversmannia* spp. (depositories of all slides in NEFU). 22 *E. atromarginata* sp. nov., paratype, slide hmx-139-2; 23 *E. bicaudata* comb. nov., slide hmx-142-2; 24 *E. exornata*, slide hmx-197-2; 25 *E. fuscifrons* comb.nov., slide hmx-103-2; 26 *E. himala* comb. nov., slide hmx-109-2; 27 *E. spiralis* sp. nov., paratype, slide hmx-172-2; 28 *E. zhangorum* sp. nov., paratype, slide hmx-124-2.

Figs 1, 2, 15, 22, 31

Epiplema bicaudata : Chu et al. 2004: 244–245, pl. 7: 8, fig. 172 (misidentification).

#### Material examined.

***Holotype*: China – Jiangxi Prov.** • ♂; Huang’ao Country, Mt Jinggang, Xiaoxidong Forest farm; 1 Jul. 2011; WC. Xie leg.; genit. prep. hmx-152-1; SYSU. ***Paratypes*: China – Jiangxi Prov.** • 1 ♂; Huang’ao Country, Mt Jinggang, Liujiaping; 4–5 Aug. 2024; HL. Han & LY. Ha & YY. Jin leg.; genit. prep. hmx-232-1; NEFU. – **Sichuan Prov.**, 1 ♀, Ya’an City, Baoxing County; 1 Aug. 2004; YD. Ren leg.; genit. prep. hmx-130-2; NKU • 2 ♀♀, Ya’an City, Baoxing County, Fengtongzhai National Nature Reserve; 3 Aug. 2004; YD. Ren leg.; genit. prep. hmx-139-2; NKU. – **Chongqing City**, 1 ♀, Simianshan National Nature Reserve, Dawopu; 4 May 2019; JJ. Fan & ZT. Wang leg.; genit. prep. hmx-173-2; NEFU.​

#### Diagnosis.

This new species is superficially similar to *E. exornata* (Eversmann, 1837) but can be distinguished from the latter by the following characters: (1) forewing: subterminal and terminal areas covered with dark-brown patches; in *E. exornata*, these areas with scattered black and brown spots or a short band; (2) male genitalia: valva slightly narrower; aedeagus slightly slenderer; in *E. exornata*, valva broader; aedeagus thicker; (3) female genitalia: corpus bursae with three signa: two small signa at base of corpus bursae and one large signum at middle of corpus bursae; *E. exornata* with only one large signum.

#### Description.

***Adult*** (Figs 1, 2, 31). Forewing length: 8–10 mm in male, 8.5–10.5 mm in female. Head: white; labial palpus up-curved, brownish white; male antennae plumose, and female filiform. Thorax: patagium and tegula white. Abdomen: covered with white scales, segments A1 and A2 with grayish white. Forewing ground color white, with dark-brown band on the costal margin in the antemedial and basal line regions; antemedial line present, a brown dot at Cu_2_-1A+2A in male, a thin rust-colored line in female; medial line absent; postmedial line double, rather dark brown, wavy, area between two lines filled with brownish red; subterminal line varies from dark brown to blackish brown; terminal and subterminal lines regions deeply gray in male, female same as ground color. Hindwing white; antemedial line rust-colored, discontinuous; postmedial line double, dark brown to dark reddish brown, wavy, prominently excurved at M_3_, area between two lines filled with light brownish red, and with outer light brownish-red line in female; marginal shade brownish red, mixed with black in posterior half, but very thin or absent before Rs in male; Rs and M_3_ with tails at outer margin; terminal line present as a small dot at M_3_-Cu_1_ in female, but in male a short, slender, distinct black strip.

***Male genitalia*** (Fig. 15). Uncus hook-shaped, apex pointed, strongly sclerotized. Tegumen triangular approximately 2× length of uncus. Gnathos sclerotized, trident-shaped, median process longest. Valva bar-shaped, covered with dense hair; sacculus arcuate, about 2/5 as long as valva; editum-costa complex bifurcate, one branch short and conical, other long and bar-shaped, and extending to the anterior of cucullus; cucullus, apex bluntly rounded; costa with weak bulge in middle; cucullus rounded, apex slightly enlarged. Juxta membranous, horseshoe-shaped, with a narrow V-shaped thin area at centre. Vinculum V-shaped. Saccus small and V-shaped. Aedeagus short and wide, slightly curved, cylindrical, with a small protuberance at middle coecum about 1/3× the length of aedeagus. Vesica membranous, with a fruit-knife-shaped cornutus, and diverticula with graniculi.

***Female genitalia*** (Fig. 22). Papillae analis conical, covered with short setae. Apophysis posterioris slender, approximately 1.5× length of apophysis anterioris. Ostium bursae like an inverted triangle, strongly sclerotized. Ductus bursae membranous, about 3/10 as long as corpus bursae. Corpus bursae membranous, densely covered with graniculi; tubular part funnel-shaped, gradually narrowing, with two small rectangular signa; globular part oval, with a large, sclerotized, durian-shaped signum.

#### Distribution.

China (Shaanxi, Jiangxi, Chongqing, Sichuan) (Fig. 40).

#### Etymology.

The species name *atromarginata* is from the Latin words “atro” and “marginata”, meaning black and margin and referring to the terminal subterminal line regions of the forewing which are covered with black patches.

#### Remarks.

Chu et al. (2004) misidentified this species as *Epiplema bicaudata* (Moore, [1868]).

### ﻿ Eversmannia bicaudata

Taxon classificationAnimaliaLepidopteraUraniidae

(Moore, [1868]) comb. nov.

F286C5B1-F31D-5CB9-8A88-A6068B0E53A1

Figs 3, 4, 16, 23, 32

Acidalia bicaudata Moore 1868: 643, pl. 33: 12. Type locality: Darjeeling, India; Type specimens: in coll. NHMUKGeometroidea-Uraniidae-Epipleminae-419-038892.
Idaea bicaudata : Cotes et al. 1888 (1887–1889): 576.
Erosia bicaudata : Butler 1889: 16.
Dirades bicaudata : Swinhoe 1894: 166.
Epiplema bicaudata : Hampson 1895: 130, fig. 71; Warren 1896: 349; Leech 1897: 186; Gielis et al. 2022: 67, pl. 67.
Epiplema fuscifrons : Inoue 1998: 81, pl. 138 fig. 4; Singh and Lekhendra 2024: 121 (2) (misidentified).

#### Material examined.

**China – Jiangxi Prov.** • 1 ♀; Jinggangshan City, Luofu Reservoir; 18 Sep. 2010; DD. Zhang & S. Zhao & B. Tong leg.; genit. prep. hmx-99-2; SYSU • 1 ♀; Shangrao City, Sanqingshan Scenic Area; 18 Jun. 2010; L. Shi leg.; SYSU. – **Xizang Autonomous Region** • 4 ♂♂; Motuo County, Beibeng Township; 31 Jul. 2018; MJ. Qi leg.; genit. prep. hmx-161-1, hmx-220-1; NKU • 3 ♂♂, 1 ♀; Motuo County, Beibeng Township, Jiagagou Bridge; 9 Jun. 2021; H. Liu leg.; genit. prep. hmx-12-1, hmx-13-1, hmx-17-1; IZCAS • 3 ♀♀; Xigaze City, Chentang Town; 25 Jun. 2021; H. Liu leg.; genit. prep. hmx-14-2, hmx-15-2, hmx-42-2; IZCAS • 2 ♂♂, 2 ♀♀; Motuo County; 5–6 Jun. 2021; HL. Han & JJ. Fan & J. Wu leg.; genit. prep. hmx-140-1, hmx-141-1, hmx-142-2; NEFU. – **Fujian Prov.** • 1 ♀; Jianou City, Wanmulin Nature Reserve; Apr. 1985; SHNU. – **Anhui Prov.** • 1 ♂; Lu’an City, Shucheng County, Wanfoshan National Forest Park; 18 Aug. 2022; JX. Wang & P. Yu leg.; genit. prep. hmx-279-1; HGNU.

#### Redescription.

***Adult*** (Figs 3, 4, 32). Forewing length: 7.5–10 mm in male, 9–12 mm in female. Head: white; labial palpus up-curved; antenna filiform. Thorax and abdomen white. Forewing ground color white; antemedial line rust-colored, discontinuous; postmedial line double, brown, waved; subterminal and terminal area with gray patches. Hindwing white; antemedial line rust-colored, discontinuous; postmedial line double, brown, wavy; marginal shade brownish red; outer margin with two short tails; terminal line present as a small dot at M_3_-Cu_1_.

***Male genitalia*** (Fig. 16). Uncus spoon-shaped, sclerotized. Tegumen triangular. Gnathos sclerotized, present as two curved blades. Valva fan-shaped, covered with dense hair; sacculus small, arcuate; editum-costa complex bifurcate, one branch short and finger-shaped, covered with short setae, other branch long and bar-shaped; cucullus rounded. Juxta membranous, horseshoe-shaped with a narrow V-shaped thin area at centre. Vinculum V-shaped. Saccus V-shaped. Aedeagus cylindrical, slightly curved. Vesica membranous, with several spine-like cornuti.

***Female genitalia*** (Fig. 23). Papillae anales hoof-shaped, covered with short setae. Ostium bursae flat, sclerotized. Ductus bursae long and slender. Corpus bursae membranous, oval, with several spine-like cornuti from the male; signum on dorsal, sclerotized, oblong.

#### Distribution.

China (Anhui, Fujian, Jiangxi, Yunnan, Xizang) (Fig. 40), Bhutan, India, Nepal, Bangladesh.

#### Remarks.

This species was formerly placed in the genus *Epiplema*. However, it shares several typical characters with *Eversmannia*: (1) forewing ground color white; postmedial line double; crossvein extremely weak; (2) hindwing white; postmedial line double; M_2_ vestigial, forming crease; (3) editum-costa complex bifurcate; (4) corpus bursae oval.

*Eversmannia bicaudata* comb. nov. shows significant differences from the type species of the genus *Epiplema*, *E. acutangularia* Herrich-Schäffer, 1855: (1) wing ground color of white; in *E. acutangularia*, grayish brown; (2) outer margin of forewing smooth; in *E. acutangularia*, dentate; (3) in male genitalia, gnathos present as two blades, editum-costa complex bifurcate; in *E. acutangularia*, gnathos and editum-costa complex absent. Consequently, we formally transfer this species to the genus *Eversmannia*.

### ﻿ Eversmannia exornata

Taxon classificationAnimaliaLepidopteraUraniidae

(Eversmann, 1837)

24610DFB-4543-55B6-A6E6-ADF2FE162A4A

Figs 5, 6, 17, 24, 33

Idaea exornata Eversmann, 1837: 65. Type locality: Kazan, Russia.
Epiplema exornataria : Herrich-Schäfer 1847: 29, fig. 418 (misspelled).
Eversmannia exornata : Staudinger 1871: 159; Fletcher 1979: 87; Dubatolov et al. 1993: 19–23; Sohn and Yen 2005: 66–67, figs 2X, 10I–K, 11D; Kishida et al. 2011: 16 pl. 1 fig. 2-1-2.
Erosia rapha Butler, 1878: 403; Butler 1879: 42, pl. 51 fig. 5.
Epiplema exornata : Seitz 1912: pl. 48i; Matsumura 1931: 941; Seitz 1933: 171–172; Bryk 1949: 28; Inoue 1982: 576, pl. 110 fig. 13.

#### Material examined.

**China – Heilongjiang Prov.** • 1 ♂; Heihe City, Pingshan Forest farm; 5–7 Jul. 2024; HL. Han & J. Wu & XR. Yang & TT. Zhao leg.; genit. prep. hmx-196-1; NEFU • 2 ♂♂, 5 ♀♀; Huma County, G331, near 12 km Bridge; 8 Jul. 2024; J. Wu & XR. Yang leg.; hmx-187-1, hmx-189-2, hmx-193-2; NEFU • 1 ♀; Huma County, Jinshan Forest farm; 8–9 Jul. 2024; HL. Han & J. Wu & XR. Yang & TT. Zhao leg.; genit. prep. hmx-197-2; NEFU • 1 ♂; Daxing’anling Prefecture, Fendou Village; 12 Jul. 2024; J. Wu & XR. Yang & TT. Zhao leg.; genit. prep. hmx-195-1; NEFU.

#### Distribution.

China (Heilongjiang) (Fig. 40), Japan, North Korea, Russia, Finland, Estonia, Latvia, Lithuania, Belarus.

#### Remarks.

Chu et al. (2004) misidentified *Dysaethria cretacae* (Butler, 1881) as this species. The DNA barcode was sequenced by Solovyev et al. (2015).

### ﻿ Eversmannia fuscifrons

Taxon classificationAnimaliaLepidopteraUraniidae

(Warren, 1896) comb. nov.

C37B3472-51D2-5F18-8F82-3FE30A2DA2FD

Figs 7, 8, 18, 25, 34

Epiplema fuscifrons Warren, 1896: 348. Type locality: Sikkim, India; type specimens: in coll. NHMUKGeometroidea-Uraniidae-Epip[eminae-41A-038893; Sohn and Yen 2005: 44–70, fig. 2W.
Epiplema bicaudata : Inoue 1998: 81, pl. 138: 3; Singh and Lekhendra 2024: 121 (2) (misidentified).

#### Material examined.

**China – Xizang Autonomous Region** • 1 ♂, 4 ♀♀; Motuo County, Deergong Village; 26 May–4 Jun. 2021; HL. Han leg.; genit. prep. hmx-216-1, hmx-103-2, hmx-104-2, hmx-199-2; NEFU • 1 ♀; Motuo County, Renqingbeng Temple; 29 Aug. 2024; YT. Fu leg.; NKU.

#### Redescription.

***Adult*** (Figs 7, 8, 34). Forewing length: 9.5 mm in male, 9–11 mm in female. Head: white mix brown; labial palpus up-curved; antenna plumose in male and filiform in female. Thorax and abdomen white. Forewing ground color white; with dark-brown band on costal margin area in antemedial and basal line regions; antemedial line brown, discontinuous; postmedial line double, brown, wavy; subterminal and terminal areas covered by dark-brown patches. Hindwing white; antemedial line brown, discontinuous; postmedial line double, with a black spot at middle; outer margin with two short tails; terminal line present as a small dot at M_3_-Cu_1_.

***Male genitalia*** (Fig. 18). Uncus hook-shaped, sclerotized. Tegumen triangular. Gnathos present as two spikes. Valva bar-shaped, covered with dense hair; sacculus small, arcuate; editum-costa complex bifurcate, one branch short and finger-shaped, covered with long hair, other branch long and bar-shaped; cucullus rounded. Juxta membranous, chestnut-shaped. Vinculum V-shaped. Saccus V-shaped. Aedeagus bow-shaped. Vesica membranous, without cornuti.

***Female genitalia*** (Fig. 25). Papilla analis hoof-shaped, covered with short setae. Ostium bursae funnel-shaped, sclerotized. Ductus bursae long and slender. Corpus bursae oval, with a star-shaped signum.

#### Distribution.

China (Xizang) (Fig. 40), India, Nepal.

#### Remarks.

Warren (1896) placed this species in the genus *Epiplema*. However, it shares several typical characters with *Eversmannia*: (1) forewing ground color white; postmedial line double; crossvein extremely weak; (2) hindwing white; antemedial line discontinuous; postmedial line double; M2 vestigial, forming crease; (3) editum-costa complex bifurcate; (4) corpus bursae oval.

*Eversmannia fuscifrons* comb. nov. can be easily distinguished from *E. acutangularia*. (1) The ground color of wings is white; in *E. acutangularia* is grayish brown. (2) The outer margin of forewing is smooth; in *E. acutangularia* it is dentate. (3) In male genitalia, the is gnathos present as two spikes and the editum-costa complex is bifurcate; in *E. acutangularia*, both the gnathos and editum-costa complex are absent. Consequently, we formally transfer this species to the genus *Eversmannia*.

The species is reported for the first time from China. The male genitalia are described for the first time. Inoue (1998) briefly compared the differences in female genitalia between *E. bicaudata* and *E. fuscifrons*.

### ﻿ Eversmannia himala

Taxon classificationAnimaliaLepidopteraUraniidae

(Butler, 1880) comb. nov.

EAEE1B00-4430-581A-9BB8-36AB2984B2F9

Figs 9, 10, 19, 26, 35

Erosia himala Butler 1880: 221. Type locality: Darjeeling, India; type specimens: in coll. NHMUKGeometroidea-Uraniidae-Epipleminae-41R-039007; Butler 1886: 47, pl. 112 fig. 9.
Epiplema himala : Hampson 1895: 131; Inoue 1998: 81, pl. 138 fig. 5.
Epiplema himala evanescens Alphéraky 1897: 139.
Epiplema evanescens : Chu et al. 2004: 243–244, pl. 7 fig. 7, fig. 171.

#### Material examined.

**China – Chongqing City** • 1 ♂; Simianshan Town, Tudiyan Station; 4–5 Aug. 2023; RT. Xu & MX. Han leg.; genit. prep. hmx-49-1; NEFU • 1 ♀; Simianshan National Nature Reserve; 29 Apr.–1 May 2019; JJ. Fan, ZT. Wang leg.; genit. prep. hmx-109-2; NEFU. – **Yunnan Prov.** • 1 ♂; Dali City, Nanjian County; 30 May 2022; RT. Xu & JJ. Fan leg.; genit. prep. hmx-50-1 NEFU. – **Sichuan Prov.** • 1 ♂, 2 ♀♀; Mabian County, Yonghong Township; 23 Jul. 2004; YD. Ren leg.; genit. prep. hmx-159-1 NKU. – Shaanxi **Prov.** • 1 ♀; Ningshan County, Xunyangba Village; 14 Aug. 2023; MJ. Qi & ZP. Chen leg.; NKU.

#### Redescription.

***Adult*** (Figs 9, 10, 35). Forewing length: 9.5–11.0 mm in male, 11.5–13.0 mm in female. Head: white; labial palpus up-curved; antenna filiform. Thorax and abdomen white. Forewing ground color white; antemedial, medial, postmedial and subterminal lines black, straight. Hindwing white, antemedial and terminal lines black, with yellow patches between the two lines; outer margin with two short tails; terminal line present as a dot at M_3_-Cu_1_.

***Male genitalia*** (Fig. 19). Uncus spoon-shaped. Gnathos present as two blades. Tegumen triangular. Valva fan-shaped, covered with dense hair; sacculus arcuate; editum-costa complex bifurcate, one branch short and conical, other branch long and bar-shaped, gradually narrowing; cucullus rounded. Juxta membranous, horseshoe-shaped with a narrow V-shaped thin area at centre. Vinculum V-shaped. Saccus long and U-shaped. Aedeagus cylindrical. Vesica membranous, with a bunch of spine-like cornuti.

***Female genitalia*** (Fig. 26). Papillae analis conical, covered with short setae. Ostium bursae flat, sclerotized. Ductus bursae membranous, short and wide. Corpus bursae membranous, with wrinkles; tubular part with oblong, serrated signum; globular part oval.

#### Distribution.

China (Hubei, Shaanxi, Chongqing, Sichuan, Yunnan) (Fig. 40), India, Nepal.

#### Remarks.

The subspecies *Epiplema himala evanescens* was upgraded to a full species by Chu et al. (2004), but they did not give an explanation for doing this. Thus, before comparing their type specimens and molecular data, we treat *E. himala evanescens* as a synonym of *E. himala*.

This species was formerly placed in the genus *Epiplema*. There are some discrepancies in the characteristic wing patterns of *E. himala* compared to typical *Eversmannia* species (the antemedial, medial, postmedial and subterminal lines of the forewing are straight), but this species still shares several typical characters with *Eversmannia*: (1) forewing crossvein extremely weak; (2) hindwing M_2_ vestigial, forming crease; (3) editum-costa complex bifurcate; (4) corpus bursae oval.

*Eversmannia himala* comb. nov. shows significant differences from *E. acutangularia*. (1) The ground color of wings is white; in *E. acutangularia*, it is grayish brown. (2) The outer margin of forewing is smooth; in *E. acutangularia*, it is dentate. (3) In the male genitalia, the editum-costa complex is bifurcate; in *E. acutangularia*, it is absent. Consequently, we formally transfer this species to the genus *Eversmannia*.

The male genitalia of this species are described for the first time.

### ﻿ Eversmannia spiralis sp. nov.

Taxon classificationAnimaliaLepidopteraUraniidae

3AE760C6-FEF6-582B-AD51-DA85AE330DC1

https://zoobank.org/2B3E24E2-3F27-48A2-AEC7-3E3DEE2BE202

Figs 11, 12, 20, 27, 36, 37

#### Material examined.

***Holotype*: China – Chongqing City** • ♂; Simianshan National Nature Reserve; 25 Apr. 2022; C. Zhang & XY. Zhang & D. Feng leg.; genit. prep. hmx-248-1; NEFU. ***Paratypes*: China – Chongqing City** • 1 ♂; same collection data as the holotype; genit. prep. hmx-250-1; NEFU • 2 ♀♀; Simianshan Town, Dawopu; 7 May 2019; JJ. Fan & ZT. Wang leg.; genit. prep. hmx-171-2, hmx-172-2; NEFU • 1 ♀; same collection data as for preceding; 4 May 2019; genit. prep. hmx-191-2; NEFU.

#### Diagnosis.

This new species can be easily distinguished from other species of the genus by the following characters: (1) forewing: with a big dark brown semicircle shaped patch at terminal area near apex; in other species only with small spots or patches near apex. (2) Male genitalia: the caecum of aedeagus strongly expanded; in other species the caecum is straight or slightly expanded. (3) Female genitalia: the ductus bursae is spiral shaped, signum absent; in other species the ductus bursae is slightly curved; signum absent.

#### Description.

***Adult*.** (Fig. 11, 12, 36, 37). Forewing length: 8.0–8.5 mm in male, 8.0–9.5 mm in female. Head: brownish mixed with white; labial palpus up-curved, dark brown; antennae filiform. Thorax: patagium and tegula white. Abdomen: covered with white scales. Forewing ground color white, with brown band on costal margin in antemedial and basal line regions; antemedial line pale brown, discontinuous; medial line absent; postmedial line present as pale-brown spots, barely visible; terminal area with a large dark-brown semicircular patch at R_4_-Cu_1_. Hindwing white; antemedial line pale brown, discontinuous; postmedial line double, pale brown, wavy, discontinuous, blurred, area between the two lines filled with light reddish brown; marginal shade brownish red, in posterior half; Rs and M_3_ with tails at outer margin; terminal line present as a small dot at M_3_-Cu_1_.

**Figures 29–39.**
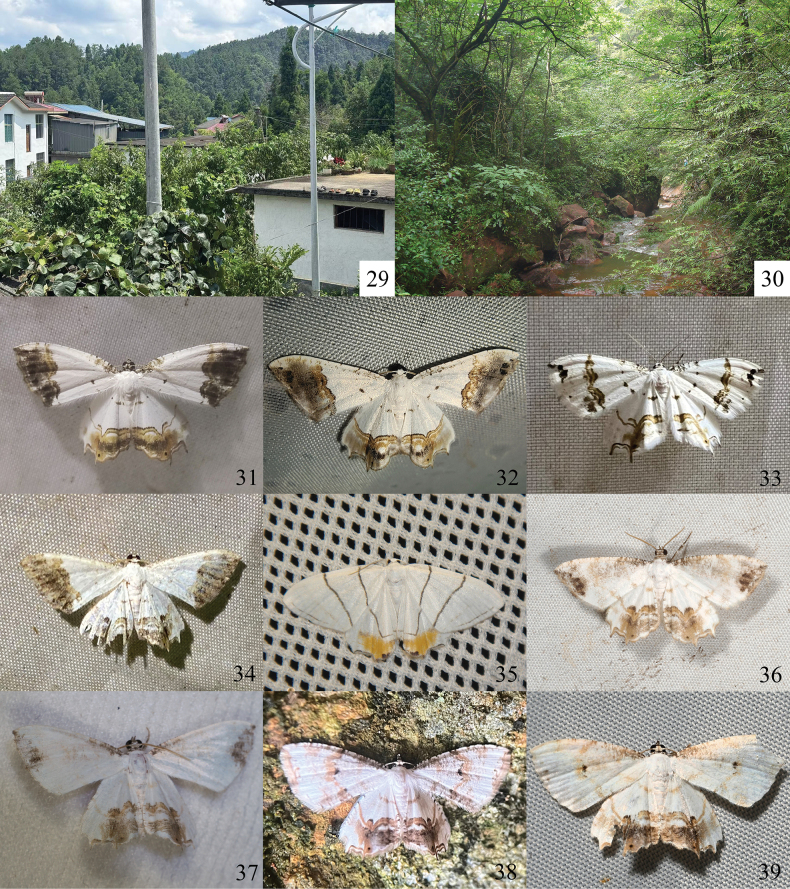
Habitats and field images of *Eversmannia* spp. 29 *E. atromarginata* sp. nov.; Prov. Jiangxi, Mt Jinggang; 30 *E. atromarginata* sp. nov., *E. spiralis* sp. nov., *E. zhangorum* sp. nov.; Chongqing City, Mt Simianshan; 31 *E. atromarginata* sp. nov.; Prov. Shaanxi, Yangxian County (Photo by XR. Yang); 32 *E. bicaudata* comb. nov.; Prov. Yunnan, Kunming City (Photo by YK. You); 33 *E. exornata*; Prov. Heilongjiang, Huma County (Photo by J. Wu); 34 *E. fuscifrons* comb. nov.; Aut. Reg. Xizang, Motuo County (Photo by YT. Fu); 35 *E. himala* comb. nov.; Chongqing City, Mt Simianshan (Photo by C. Zhang); 36, 37 *E. spiralis* sp. nov.; Prov. Guizhou, Tongren City, Mt Fanjingshan; Prov. Zhejiang, Hangzhou City, Mt Tianmushan (Photo by Z. Peng); 38, 39 *E. zhangorum* sp. nov.; Prov. Hunan, Liuyang City, Mt Daweishan; Prov. Zhejiang, Ningbo City, Longguan Township (Photo by WX. Jiang and YJ. Hu).

***Male genitalia*** (Fig. 20). Uncus long hook-shaped, apex pointed, strongly sclerotized. Gnathos sclerotized, present as two spikes. Tegumen triangular, approximately 1.5× length of uncus. Valva bar-shaped, covered with dense hair; sacculus arcuate, about as 1/4 long as valva; editum-costa complex bifurcate, one branch short, finger-shaped and covered with long hair, other branch long, bar-shaped, and extending to anterior of cucullus; costa extended in front part; cucullus rounded, slightly enlarged apical. Juxta membranous, water-chestnut-shaped. Vinculum broad V-shaped. Saccus V-shaped. Aedeagus bow-shaped; caecum strongly expanded, about 1/2× length of aedeagus. Vesica membranous, with bunch of spine-shaped cornutus, and diverticula with graniculi.

***Female genitalia*** (Fig. 27). Papillae anales hoof-shaped, densely covered with short setae. Apophysis posterioris slender, approximately 1.5× length of apophysis anterioris. Ostium bursae flat, slightly sclerotized. Ductus bursae slender, membranous, spiral, about as long as corpus bursae. Corpus bursae membranous, long-drop-shaped, moderately curved, slightly sclerotized at the base, signum absent.

#### Distribution.

**Figure 40.**
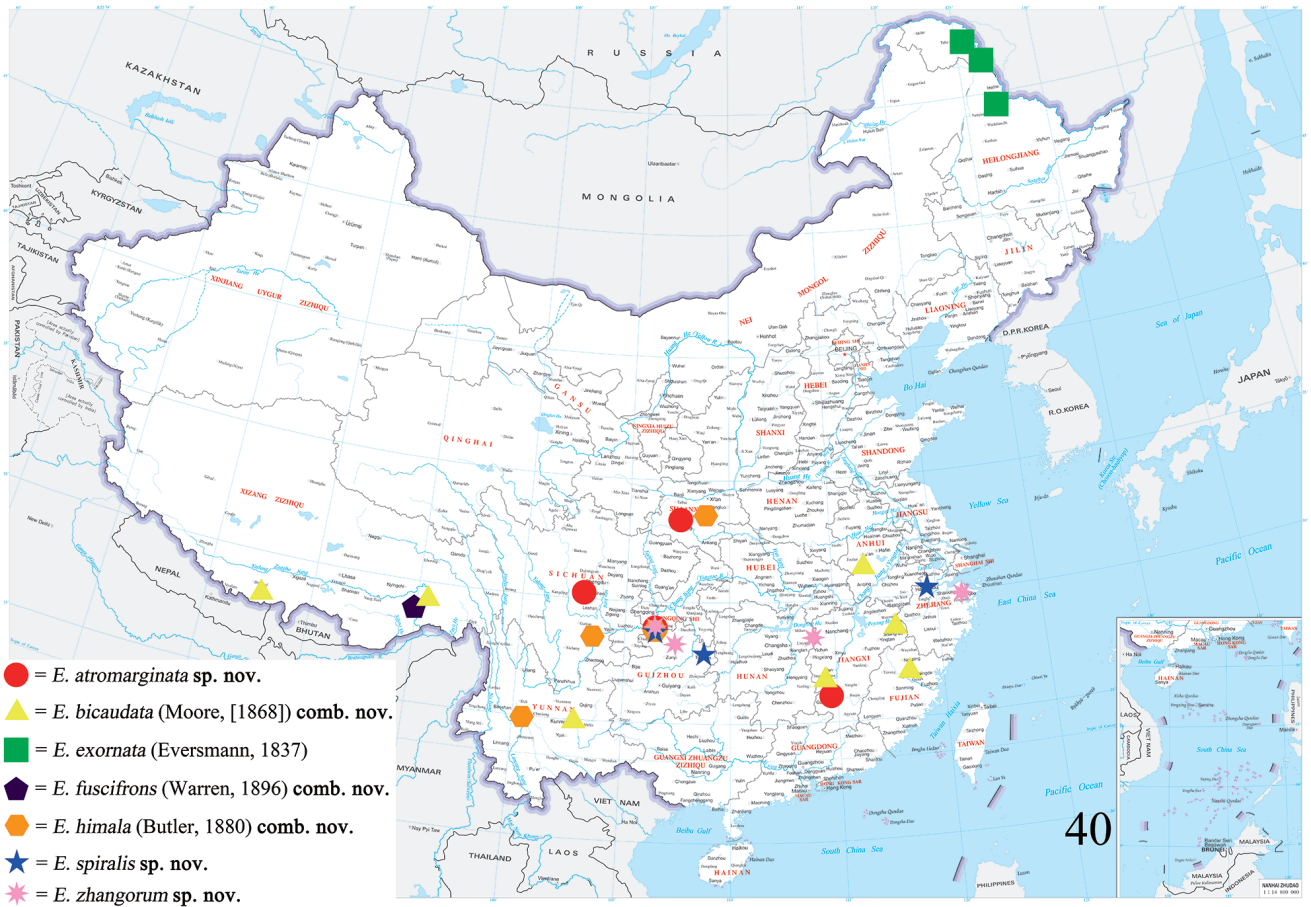
Distribution map of *Eversmannia* spp. in China.

China (Zhejiang, Chongqing, Guizhou) (Fig. 40).

#### Etymology.

The species name is from the Latin word “spiralis”, meaning spiraling, referring to the spiral corpus bursae.

### ﻿ Eversmannia zhangorum sp. nov.

Taxon classificationAnimaliaLepidopteraUraniidae

123EC952-8D17-53F9-BC42-E95E696939BF

https://zoobank.org/92A7DBB7-2260-4720-B39F-98A0A13C47C8

Figs 13, 14, 21, 28, 38, 39

#### Material examined.

***Holotype*: China – Chongqing City** • ♂; Simianshan National Nature Reserve; 19 Aug. 2021; C. Zhang & XY. Zhang & L. Luo leg.; genit. prep. hmx-249-1: NEFU. ***Paratype*: China – Guizhou Prov.** • 1 ♀; Zunyi City, Shierbeihou Scenic Spot; 3–5 Aug. 2020; HL. Han & J. Wu leg.; genit. prep. hmx-124-2; NEFU.

#### Diagnosis.

This new species is similar to *E. fuscifrons* but can be distinguished from the latter by the following characters: (1) forewing: the postmedial line with a black spot at the middle; in *E. fuscifrons*, postmedial line without spot; (2) male genitalia: gnathos spear-shaped; in *E. fuscifrons*, gnathos spine-shaped; (3) female genitalia: signum reniform with one edge serrate; in *E. fuscifrons*, signum star-shaped.

#### Description.

***Adult*** (Fig. 13, 14, 38, 39). Forewing length: 8.5 mm in male, 9.0 mm in female. Head: white; labial palpus up-curved, brown; antennae filiform. Thorax: patagium and tegula white. Abdomen: covered with white scales. Forewing: ground color white, with dark-brown patches on costal margin area; antemedial line pale brown, discontinuous; medial line absent; postmedial line pale brown, discontinuous, outward-curved on costal margin region, then inwardly curved to inner margin, with a large black spot at middle. Hindwing white; antemedial line pale brown, discontinuous; postmedial line double, rust-colored, wavy, prominently excurved at M_3_, inner side with a crescent-shaped spot at middle; marginal shade brownish red, mixed with dark brown in posterior half; Rs and M_3_ with tails at outer margin; terminal line present as a small dot at M_3_-Cu_1_.

***Male genitalia*** (Fig. 21). Uncus long, hook-shaped, strongly sclerotized. Tegumen triangular, approximately equal to length of uncus. Gnathos sclerotized, spear-shaped, present as two blades, apex pointed. Valva bar-shaped, covered with dense hair; sacculus arcuate, about 1/3 as long as valva; editum-costa complex bifurcate, one branch short and finger-shaped, apex rounded, covered with hair, other branch long and bar-shaped and extending before cucullus; costa with a small bulge in middle; cucullus rounded, apex slightly shrunken. Juxta missing. Vinculum broad and U-shaped. Saccus small and V-shaped. Aedeagus cylindrical, distally curved. Vesica membranous, cornutus absent.

***Female genitalia*** (Fig. 28). Papilla analis hoof-shaped, densely covered with short setae. Apophysis posterioris slender, approximately 2× the length of apophysis anterioris. Ostium bursae funnel-shaped, sclerotized. Ductus bursae slender, membranous, approximately 1.5× length of corpus bursae. Corpus bursae membranous, oval, densely covered with graniculi, signum reniform, with serrate on one edge.

#### Distribution.

China (Zhejiang, Hunan, Chongqing, Guizhou) (Fig. 40).

#### Etymology.

This species is dedicated to Mr Chao Zhang and Ms Xin-Yu Zhang, who collected the holotype.

## Supplementary Material

XML Treatment for Eversmannia

XML Treatment for Eversmannia atromarginata

XML Treatment for Eversmannia bicaudata

XML Treatment for Eversmannia exornata

XML Treatment for Eversmannia fuscifrons

XML Treatment for Eversmannia himala

XML Treatment for Eversmannia spiralis

XML Treatment for Eversmannia zhangorum

## ﻿Additional information

### Use of AI

No use of AI was reported.

### Author ORCIDs

Ming-Xu Han https://orcid.org/0009-0002-9252-5981

Hui-Lin Han https://orcid.org/0000-0002-2045-6182

### Data availability

All of the data that support the findings of this study are available in the main text.
